# Supplementary material for: The interactome of intact mitochondria by cross-linking mass spectrometry provides evidence for coexisting respiratory supercomplexes
Source: Mol Cell Proteomics. 2017 Dec 8;17(2):216–32. doi: 10.1074/mcp.RA117.000470 (PMC5795388; doi:10.1074/mcp.RA117.000470)
Supplement: Supplemental Data [file supp_RA117.000470_133922_0_supp_23978_fzffwf.zip › spectra_annotation/mito_DR_spectra_annotation/316-1-1-1-7-1.pdf]

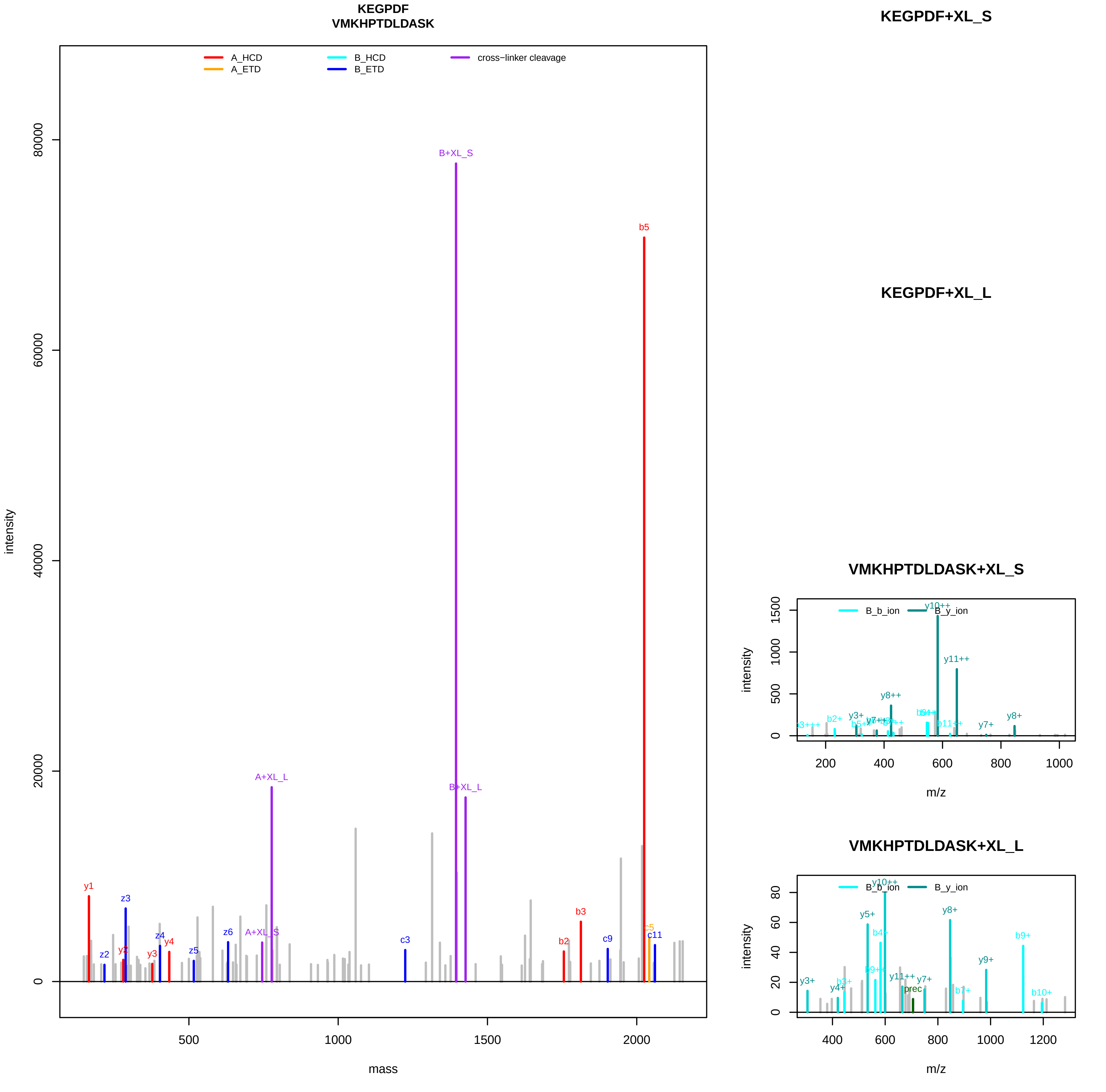
KEGPDF
VMKHPTDLDASK
A_HCD
A_ETD
B_HCD
B_ETD
cross−linker cleavage
B+XL_S
b5
A+XL_L
B+XL_L
A+XL_S
y1
y2
y3
y4
z2
z3
z4
z5
z6
c3
b2
b3
c9
c5
c11
intensity
mass
KEGPDF+XL_S
KEGPDF+XL_L
VMKHPTDLDASK+XL_S
B_b_ion
B_y_ion
y10++
y11++
y8++
y3+
y7+
y8+
b2+
b11++
m/z
VMKHPTDLDASK+XL_L
y5+
b4+
y8+
b9+
y9+
y3+
y4+
b3+
y11++
y7+
prec
b7+
b10+
